# A model of *Plasmodium vivax* concealment based on *Plasmodium cynomolgi* infections in *Macaca mulatta*

**DOI:** 10.1186/s12936-017-2008-4

**Published:** 2017-09-18

**Authors:** Luis L. Fonseca, Chester J. Joyner, MaHPIC Consortium, Mary R. Galinski, Eberhard O. Voit

**Affiliations:** 1 The Wallace H. Coulter Department of Biomedical Engineering, Georgia Institute of Technology and Emory University, 950 Atlantic Drive, Suite 2115, Atlanta, GA 30332-2000 USA; 2 International Center for Malaria Research, Education and Development, Emory Vaccine Center, Yerkes National Primate Research Center, Emory University, 954 Gatewood Road, Atlanta, GA 30329 USA; 3 Division of Infectious Diseases, Department of Medicine, Emory University, Atlanta, GA USA; 4 Malaria Host-Pathogen Interaction Center, Atlanta, GA USA

**Keywords:** Host–pathogen interactions, Malaria, Mathematical model, Parasite dynamics, *Plasmodium falciparum*, Sequestration, Systems biology

## Abstract

**Background:**

*Plasmodium vivax* can cause severe malaria. The total parasite biomass during infections is correlated with the severity of disease but not necessarily quantified accurately by microscopy. This finding has raised the question whether there could be sub-populations of parasites that are not observed in peripheral blood smears but continue to contribute to the increase in parasite numbers that drive pathogenesis. Non-human primate infection models utilizing the closely related simian malaria parasite *Plasmodium cynomolgi* hold the potential for quantifying the magnitude of possibly unobserved infected red blood cell (iRBC) populations and determining how the presence of this hidden reservoir correlates with disease severity.

**Methods:**

Time series data tracking the longitudinal development of parasitaemia in five *Macaca mulatta* infected with *P. cynomolgi* were used to design a computational model quantifying iRBCs that circulate in the blood versus those that are not detectable and are termed here as ‘concealed’. This terminology is proposed to distinguish such observations from the deep vascular and widespread ‘sequestration’ of *Plasmodium falciparum* iRBCs, which is governed by distinctly different molecular mechanisms.

**Results:**

The computational model presented here clearly demonstrates that the observed growth data of iRBC populations are not consistent with the known biology and blood-stage cycle of *P. cynomolgi*. However, the discrepancies can be resolved when a sub-population of concealed iRBCs is taken into account. The model suggests that the early growth of a hidden parasite sub-population has the potential to drive disease. As an alternative, the data could be explained by the sequential release of merozoites from the liver over a number of days, but this scenario seems less likely.

**Conclusions:**

Concealment of a non-circulating iRBC sub-population during *P. cynomolgi* infection of *M. mulatta* is an important aspect of this successful host–pathogen relationship. The data also support the likelihood that a sub-population of iRBCs of *P. vivax* has a comparable means to become withdrawn from the peripheral circulation. This inference has implications for understanding vivax biology and pathogenesis and stresses the importance of considering a concealed parasite reservoir with regard to vivax epidemiology and the quantification and treatment of *P. vivax* infections.

**Electronic supplementary material:**

The online version of this article (doi:10.1186/s12936-017-2008-4) contains supplementary material, which is available to authorized users.

## Background

*Plasmodium vivax* is a major infectious disease agent that causes substantial morbidity in communities around the world where it is endemic, and about 2.5 billion people live at risk of infection and possible death [1–3]. Most critically, this species also has a dormant stage in the liver that can activate and cause new blood infections known as relapse infections (reviewed in [4–6]). Together, primary and relapsing infections permit increased opportunities for spreading the disease via *Anopheles* mosquito vectors, which serve as the definitive host. The relapses, characterized through experiments using the closely related non-human primate (NHP) infection model of *Plasmodium cynomolgi* and *Macaca mulatta* (rhesus macaque), may not cause clinical illness but serve as a reservoir to ensure transmission [7]. An improved understanding of the primary and relapse blood-stage infections of these species in their respective hosts is critical for developing effective *Plasmodium vivax* elimination strategies [8]. Moreover, improved knowledge about the blood-stage forms is essential to understanding disease pathogenesis, as are underlying factors that may cause severe vivax malaria (reviewed in [9]). The mechanisms of infected red blood cell (iRBC) biology of these two *Plasmodium* species, host-parasite interactions, possible adhesion and resulting pathology remain largely unexplored.

Malaria caused by *P. vivax* and *P. cynomolgi* is characterized by the circulation of iRBCs containing the various forms of the parasite’s blood-stage development, including the asexual ring, trophozoite and schizont forms, as well as the sexual gametocyte forms. Recent results by Barber and colleagues [10, 11] and others (reviewed in [2]) refer to a hidden population of vivax parasites that may be part of the total biomass and important for causing severe disease. In fact, Baird wrote, “Parasites concealed within the marrow and spleen, and certainly hypnozoites in the liver, may all represent substantially larger proportions of the populations surveyed. The true prevalence of *P. vivax* in zones of endemicity may be considerably higher than that suggested by mass blood film examinations” [2].

Notably, *P. vivax* and *P. cynomolgi* lack counterparts of the large variant antigen family characteristic of *Plasmodium falciparum*, which causes iRBC adhesion to endothelium receptors and leads to a phenomenon known as sequestration. Sequestration in this context can be defined as the detainment of the vast majority of asexual stage iRBCs from the peripheral circulation as they are maturing into trophozoites and schizonts, which can contribute to the occlusion of post-capillary venules in various tissues and organs with associated pathology (reviewed in [12, 13]). While sequestration of *P. falciparum* iRBCs has been recognized for over a century, and is obvious from the examination of peripheral blood smears [14], details of the crucial underlying molecular mechanisms of cytoadherence have been emerging over only the past 25 years. Of particular importance, the erythrocyte membrane protein-1 (EMP-1) variant antigens, encoded by the large *var* multi-gene family, are expressed at the surface of *P. falciparum* iRBCs. *P. falciparum* EMP-1 is typically ~300 kDa and encoded by about 60 *var* gene family members. It is characterized by a large extracellular domain comprised of cytoadherent modules known to adhere to various host receptors, notably on endothelial cells in the small capillary venules, and possibly also on uninfected RBCs, resulting in rosette formation (reviewed in [15–19]. On the host side, several proteins serving as receptors for *P. falciparum* iRBCs have been identified on endothelial cells, including, among others, CD36, thrombospondin, intercellular adhesion molecule-1, and chondroitin sulfate. It has also been observed that the spleen somehow modulates the parasite’s expressed proteins at the iRBC outer membrane, and that sequestration is reduced in splenectomized animals [20, 21]. This is comparable to landmark observations in *Plasmodium knowlesi* infection of rhesus macaques (reviewed in [22]).

In stark contrast to *P. falciparum* and *P. knowlesi*, *P. vivax* and *P. cynomolgi* do not possess the *var* multi-gene family. However, *P. vivax* has a different large multi-gene family comprised of many small *vir* genes encoding VIR proteins [23], and comparable genes (i.e., the *cyir* family) are present in *P. cynomolgi* [24]. In fact, as many as 1200 and 1300 of these genes have now been found in *P. vivax* and *P. cynomolgi*, respectively [25, 26], and they are now known more broadly across various species (including those infecting rodents) as *pir* genes expressing PIR proteins [27–29]. VIR proteins have been implicated as possible adhesins of iRBCs, and provide a possible explanation of local pathology in tissues and organs [30]; however, research in this area is complex and preliminary, and other proteins or families of proteins may have corresponding roles. Although the *vir* gene family is large and diverse, and the proteins are small and difficult to characterize, the expression of a sub-set of VIR proteins was confirmed recently in the proteomes of *P. vivax* trophozoites and schizonts [31, 32], kindling further inquiry into their functions in the context of host–pathogen interactions for the survival of this species. The highly predominant PHIST/CVC-81_95_ protein [28, 33, 34] may also play a role, among other known and yet to be identified candidates from the *P. vivax* and *P. cynomolgi* parasite’s complex genome encoding many hypothetical proteins [26, 35]. Moreover, recent ex vivo experiments showed that *P. vivax* merozoites have a strong preference for invading the immature CD71^+^ sub-population of reticulocytes, which are typically in the bone marrow, thus raising the possibility that the bone marrow may serve to house a sub-population of *P. vivax* iRBCs [36].

The work presented in this article uses the rhesus macaque—*P. cynomolgi* animal model of vivax malaria to investigate an apparently hidden population of *P. vivax*, which begins to develop early during the blood-stage infection [10, 11]. Specifically, it proposes a mathematical model to define and characterize the dynamics of iRBC sub-populations that represent circulating and *concealed* asexual stage parasites, respectively. The term *concealment* is adopted here from [2] to explain the presence of such a hidden sub-population and to clearly distinguish the putative processes and mechanisms that may be involved from those utilized by *P. falciparum*, where the vast majority of maturing asexual stage iRBCs cytoadhere and become *sequestered* in the microvasculature.

Mathematical modelling has been used to study cell populations within blood (e.g., [37–39]), and some models have specifically targeted the dynamics of RBCs in malaria (e.g., [40–48]). In fact, a few models have addressed the issue of parasite sequestration. However, their focus was on estimating typical percentages of iRBCs outside of the circulation in murine parasites such as *Plasmodium berghei,* or in *P. falciparum*, and to assess the speculation that the severity of disease is associated with the total parasite load [49–55]. The goal here is distinct in this study’s attempt to characterize the likelihood and degree of the temporary removal from circulation of sub-populations of *P. cynomolgi* or *P. vivax* iRBCs, especially early in an infection, as an evolved mechanism of these species and perhaps others that ensures survival in the host. The implications are broader, as discussed, with regard to the proposed concealment of a large biomass of *P. vivax* iRBCs in the haemopoietic tissues [2].

## Methods

### Data

Details of the experimental infections of five rhesus macaques, data acquisition prior to and over a 100-day infection period, and raw data analysis were recently described in Joyner et al. [7]. The data from this experiment have been deposited to PlasmoDB [56]. Selected highlights of the experimental design, clinical presentation of the animals, and data collection pertinent to the current modelling study are summarized in the Additional file 1.

### Model

**Fig. 1:**
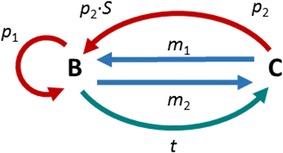
Diagram of the model accounting for infected red blood cells (iRBCs) in the bloodstream (*B*) or in concealment (*C*). Each iRBC releases a brood of *S* merozoites, which enter circulation where they infect uRBCs. Parasites may migrate between bloodstream and concealment with rates *m*_1_ and *m*_2_, respectively. Furthermore, there is constant net transport of cells into concealment with rate *t*. The parasites proliferate with rates *p*_1_ and *p*_2_

The model contains pools representing iRBCs in the bloodstream (*B*) and in concealment (*C*) (Fig. 1). The parasites released by any iRBC migrate through the blood and infect uninfected RBCs (uRBCs). The transport into concealment consists of two processes. One is directly proportional to the number of iRBCs in *B*, whereas the other is constant and thus independent of the number of iRBCs; in addition, iRBCs may migrate between the two compartments in both directions. A slightly different model variant is described in the Additional file 1.

The model was implemented with the two ordinary differential equations as shown in Eq. (1).

$$\dot{B} = p_{1} B + p_{2} \cdot S \cdot C + m_{1} C - t - m_{2} B$$

1 $$\dot{C} = t + m_{2} B - p_{2} C - m_{1} C$$

Here, the left-hand sides represent changes over time, and all rate constants are positive. The rates *m*_1_, *m*_2_, and *t*, are considered parameters that are to be estimated from the data; their estimation is discussed in the Additional file 1, along with a discussion of the initial values *B* (*T*_0_) and *C* (*T*_0_) and the rates of the proliferation processes *p*_1_ and *p*_2_.

Experience with *P. cynomolgi* indicates that the average brood size of merozoites per iRBC is typically restricted to a range between 14 and 20, with a most likely value of 16, as discussed before. Thus, all other parameters in the model were fitted three times, with *S* fixed at 14, 16, or 20 [57].

## Results

**Fig. 2:**
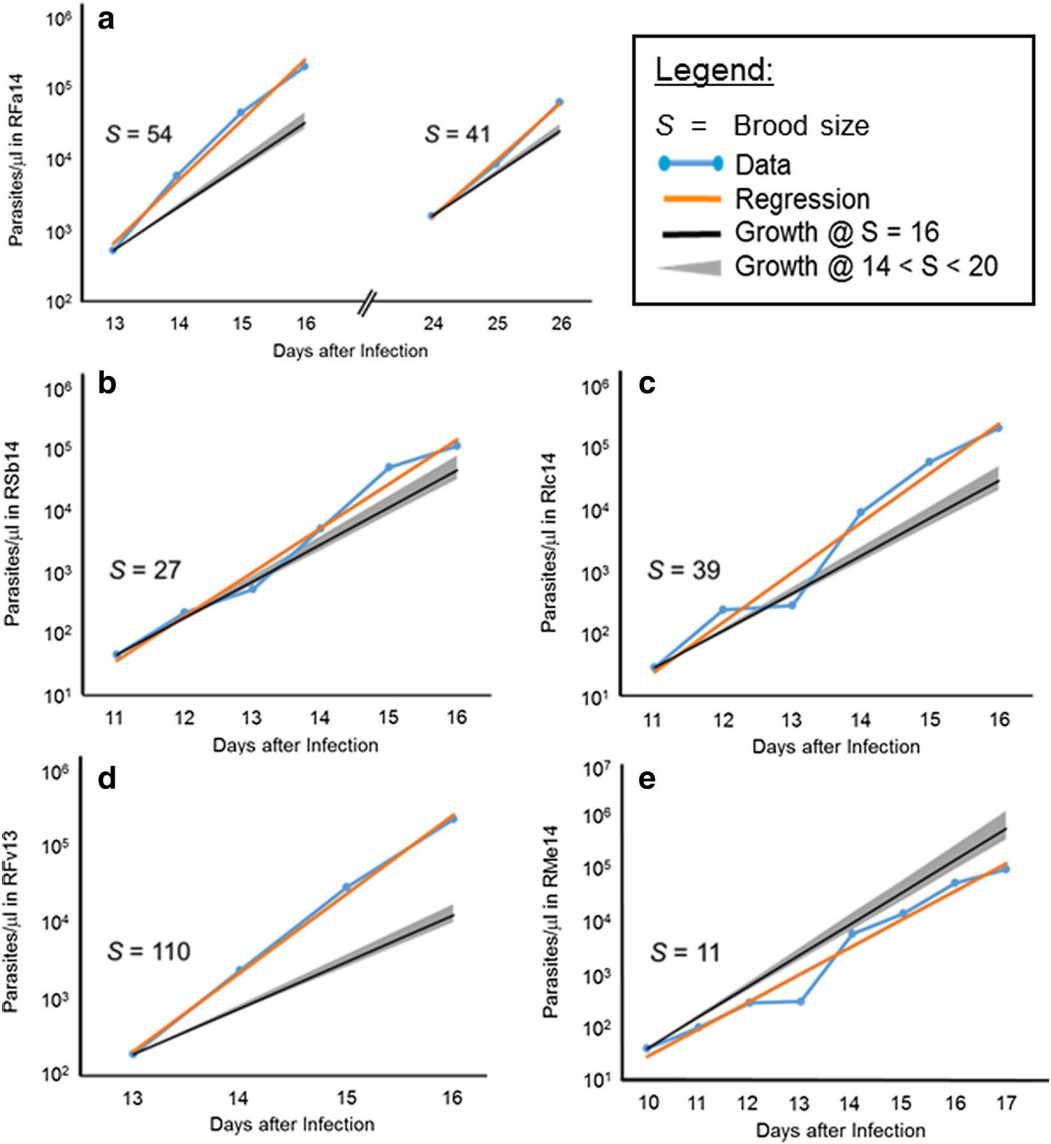
Growth of *Plasmodium cynomolgi* populations during five macaque infections. The *light blue dots* (for better visibility connected with *lines*) represent parasitaemia data (Y-axis, parasites/µl) reported by Joyner et al. [7] on specific days post-infection as noted on the X-axis. At earlier time points, no iRBCs were observed in blood smears. The *orange lines* show the best exponential fits corresponding to brood size *S*. The *slim grey cones* indicate growth corresponding to reported brood size ranges between 14 and 20, with the *overlapping black lines* corresponding to an average brood size of 16. **a** Typical growth trend of a parasite population, observed during a primary infection and a recrudescence subsequent to sub-curative treatment; the computed average brood sizes *S* in this case are 54 and 41, respectively. **b**–**d** According to the best exponential fits to the observed population sizes during the primary infection, the brood sizes *S* in these* panels* are 27, 39, and 110, respectively. **e** This population shows much slower growth, with an average viable brood size *S* of about 11, which is lower than the observed range and could be explained by reduced parasite efficiency. All monkeys had one or two relapses with lower parasitaemia levels and inferred brood sizes of: 7 (RFa14); 6 and 8 (RSb14); 10 and 8 (RIc14); 7 (RFa14); and 17 (RMe14)

The motivation for the study came from blood samples strategically acquired before and during the infections of five *M. mulatta* with *P. cynomolgi* [7], which permitted a straightforward analysis of the population growth of the blood-stage parasites. At first glance, the results looked unremarkable, especially when drawn in logarithmic plots, which clearly seem to identify the growth process as very close to exponential (Fig. 2). However, when the process was scrutinized more closely, the growth rates necessary to match the experimentally determined growth curves turned out to be much higher in four out of five animals than is biologically reasonable, based on knowledge of the species [58]. Namely, the typical number of *P. cynomolgi* merozoites released from mature schizonts has been determined to be about 16 or 18, with lower and upper limits of 14 and 20, respectively [57].

The observed trends shown in Fig. 2 were analysed, and the growth rates were computed and used to infer the average number of merozoites released necessary to fit the observations. For one of the five monkeys assessed (RMe14), the effective brood size was computed to be about 11. This size is less than what is considered typical, but it is close to the expected number of merozoites per iRBC. However, data from the remaining four monkeys suggested brood sizes between 30 and over 100, which are biologically infeasible given the limitations of the size of the RBC. Thus, this modelling study was designed to determine whether the observed growth curves could be explained if one accounts for parasites that are concealed and, therefore, not circulating and not taken into account when determining parasitaemia by microscopy.

All monkeys in the study had one or two relapses with lower parasitaemia levels and growth characteristics that suggested much smaller brood sizes than for the primary infection, with the exception of RMe14, whose parasite growth during relapse suggested a brood size of 17. For RSb14, RIc14 and RFa14, the inferred brood sizes were 6 and 8, 10 and 8, and 7, respectively. The much smaller brood sizes during relapses indicate the substantial effect of the immune system.

### Growth curves accounting for concealment of *Plasmodium cynomolgi* infected red blood cells

**Fig. 3:**
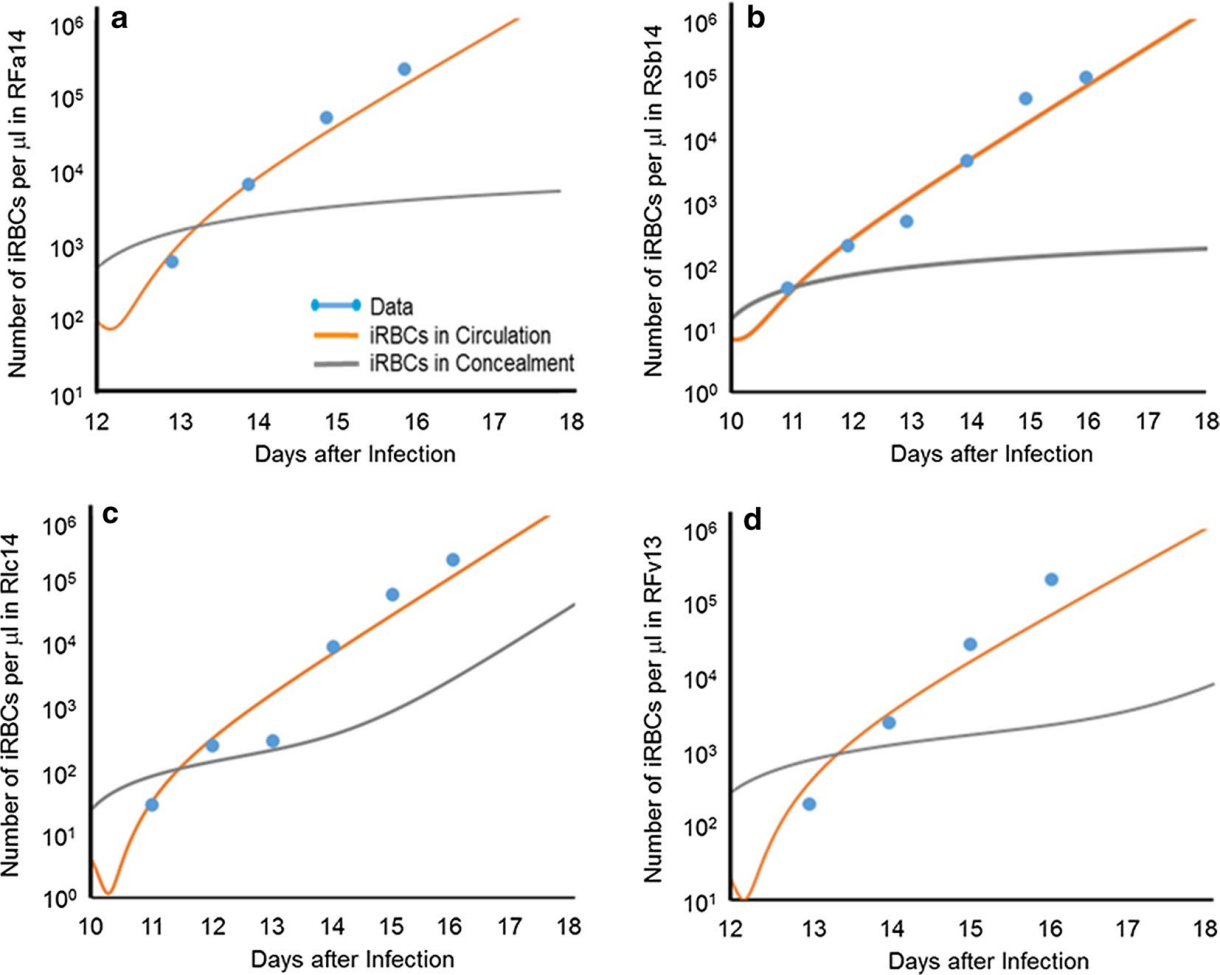
Growth of infected red blood cell (iRBC) populations in four monkeys, namely: **a** RFa14, **b** Rsb14, **c** RIc14, and **d** RFv13. The data (*dots*) are well fitted by the modelled iRBCs in the blood (*orange line*). The pool of concealed iRBCs (*grey line*) is initially very important, but its contribution to the overall iRBC population becomes less significant later. Monkey RMe14 was not analysed, as its iRBC growth characteristics could be explained without concealment

Without concealment, exponential functions fit the data very well (Fig. 2); however, only with biologically unreasonable brood sizes for the four animals with fast growth. Accounting for concealment in the model, the discrepancy can be resolved. Figure 3 exhibits the results of these analyses. Parameter values for these results are presented in the Additional file 1.

In all four cases of apparently unreasonable brood sizes, the simulations start 1 day before the first iRBCs were observed in blood smears. The initial value for *B* (iRBCs in circulation) was computed for each monkey from a linear regression of the data points in a logarithmic plot (Fig. 2), which was extrapolated back to day 11 post-infection with sporozoites [7]. The initial value for *C* (iRBCs in concealment) at this time point was considered a free parameter that was optimized along with the transport rates for a merozoite brood size of 14, 16, or 20. At the beginning of each simulation, pool *B* temporarily decreases, because iRBCs move into concealment, causing *C* to increase. However, as Fig. 3 clearly shows, this trend is rapidly overcome by proliferation, and pool *B* grows, ultimately approaching the specified growth rate. Pool *C* also grows, but in three of the four cases more slowly. At the peaks of the primary parasitaemias (between days 18 and 20), the size of the concealment pool in each case is only a small fraction of the size of pool *B*. Thus, the pool of concealed iRBCs is initially very important, but its significance decreases later. One should also note that the antibody response against the parasites starts to occur at about this time and begins to neutralize parasites, so that fewer merozoites invade RBCs.

### Growth during the pre-patent blood stage

**Fig. 4:**
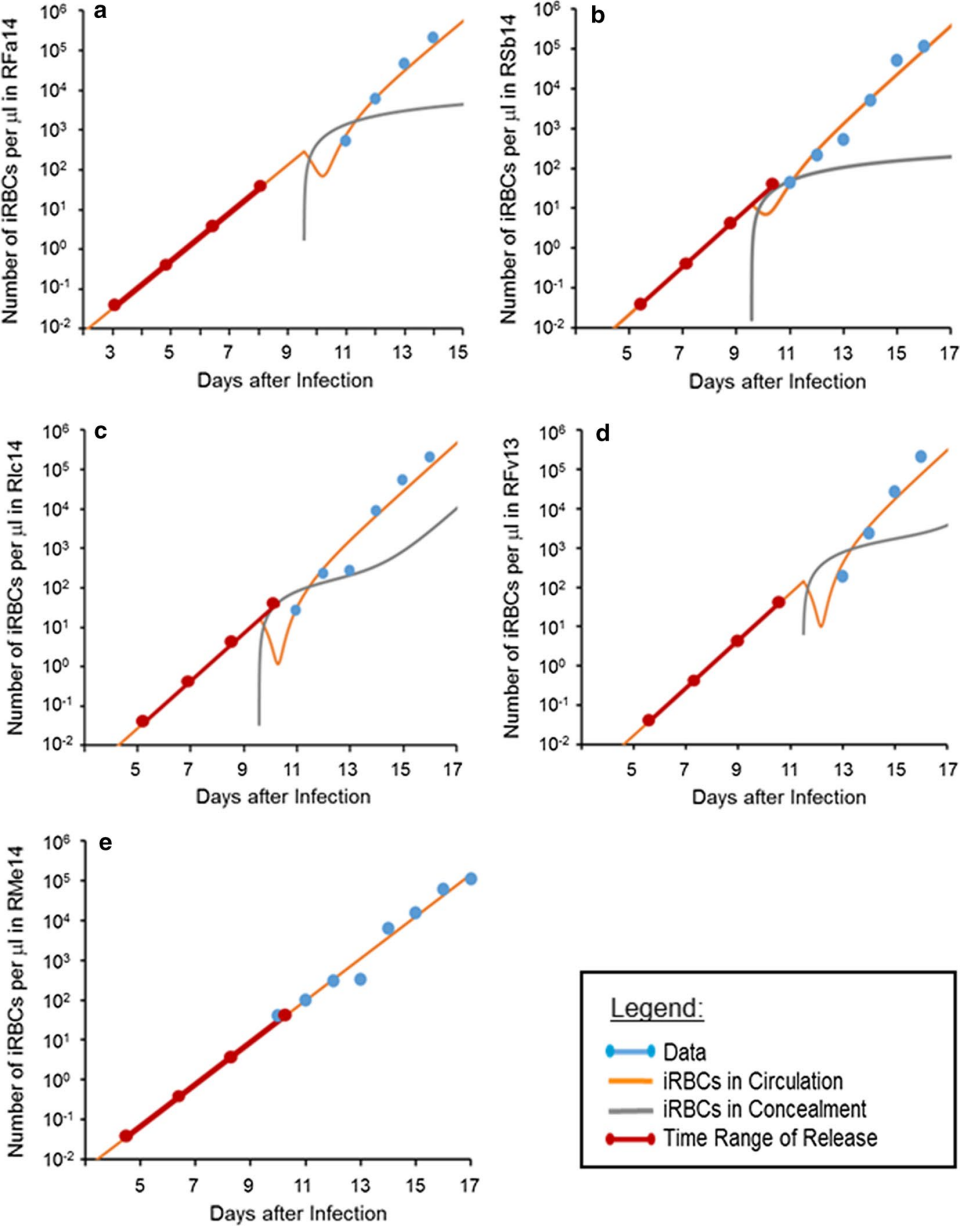
Backward extrapolation of the time trends in infected red blood cell (iRBC) populations for the five monkeys used in this study, namely: **a** RFa14, **b** Rsb14, **c **RIc14, **d** RFv13, and **e** RMe14. The data (*blue dots*) are well fitted by the modelled iRBCs in the blood (*orange line*). The pool of concealed iRBCs (*grey line*) is initially important, but its contribution to the total iRBC population becomes less significant later. The *thick burgundy line* indicates the time range of merozoite release from the liver, with *dots* representing, from *left* to *right*, scenarios where 1, 10, 100, or 1000 sporozoites, respectively, were successful in invading the liver and leading to the production of 20,000 merozoites each. As an example, if 100 sporozoites are successful, release from the liver is predicted to occur about 10 days after infection, except for **a** (RFa14 infection), where it is predicted to happen at about day 8

The model for each monkey was extrapolated back towards earlier days of infection (see “Methods”). This extrapolation was achieved by computing the exponential growth function with a typical rate corresponding to a brood size of 14, 16 or 20. The results are coarse predictions of the growth of an iRBC population below the detection limit, and, in the process, allows for the approximate determination of the likely time range of merozoite release from the liver. Figure 4 depicts these extrapolations for all five monkeys, including RMe14, whose observed growth corresponds to a reasonable average brood size. The approximate determination of the time point of release of merozoites from the liver depends on several assumptions. First, the number of merozoites released by a mature liver-stage schizont is assumed to be between 5000 and 40,000 for a single infected hepatocyte, with a typical average of about 20,000 for *P. vivax*, and presumably for *P. cynomolgi* (J W Barnwell, pers. comm). Second, in the experiments used here, 2000 sporozoites were injected. Many of these will not result in merozoites early in the primary blood-stage infection as they either become hypnozoites, do not invade the liver or fail to mature. Finally, the numbers have to be scaled to blood volume, which is assumed to be 500 ml, based on an 8-kg animal having approximately 60–70 ml blood per kg [58, 59]. Thus, assuming a range between 1 and 1000 sporozoites that are ultimately leading to 20,000 merozoites each, one obtains a time range during which this release occurred. This time range is shown in Fig. 4 with thick burgundy lines, where dots mark results from assuming 1, 10, 100, or 1000 sporozoites to have been viable, entered into schizogony after invasion, and produced merozoites that are released into the blood to initiate the blood-stage infection. For the assumption of 10 or 100 sporozoites that underwent these processes, the release seems to happen about 7–9 days after infection and, thereby, 2–5 days before parasites were detected in blood smears. This timing of release is consistent with an 8-day liver-stage maturation period for this species [57]. Whether, 10,000, 20,000 or 30,000 merozoites are used in these simulations does not affect the locations of the dots much, as the results are exhibited on a log-10 scale.

## Discussion

Until this Century, it was generally thought that *P. falciparum* was the only human malaria parasite able to ‘hide’ its maturing asexual stage iRBCs, presumably to prevent removal by the spleen, and through processes that have come to be known as cytoadherence and sequestration. The main reasons for this uniqueness assumption were: (1) unlike *P. falciparum*, all asexual blood-stage forms for every other human malaria parasite species (*P. vivax*, *Plasmodium malariae* and *Plasmodium ovale*, and also the zoonotic species *P. knowlesi*) are observable on peripheral blood smears; and, (2) there has been a lack of known confirmed receptor ligand interactions responsible for such processes in other parasite species, at least, akin to PfEMP-1 and its various receptors (reviewed in [17–19]. Nevertheless, intuitively, a hiding strategy would be advantageous for the growth of a new parasite population within a host; thus it should not be surprising for other *Plasmodium* species to have developed alternative approaches for survival, albeit involving different molecular mechanisms.

Indeed, recent investigations using samples collected from vivax malaria patients have demonstrated that determining parasitaemia with peripheral blood either by smear or molecular techniques can underestimate the total *P. vivax* parasite biomass in a given individual [10]. This work and other prior studies (reviewed in [2]) have led to the speculation that *P. vivax* may also possess a means to escape the circulation in an attempt to avoid removal by the host, or simply have developed a niche to grow and multiply in haemopoietic tissues [2]. In the current study, analysing the population growth of the simian malaria parasite *P. cynomolgi* in rhesus macaques revealed that the growth rates required to fit the data were much too high to be biologically feasible for four out of five rhesus macaques studied. Specifically, the analysis determined that each iRBC observed in the periphery would have to produce approximately 30–110 merozoites in each generation to achieve the parasitaemia determined by microscopy during the infection. Through mathematical modelling, this inconsistency became explainable and was completely resolved by postulating the existence of a process termed here as *concealment*. This term was used to distinguish this process from the distinct deep vascular sequestration of iRBCs during infections with *P. falciparum,* as well as simian species such as *Plasmodium coatneyi* or *Plasmodium fragile* that are known to have this characteristic (reviewed in [22]). Accounting for concealment yielded excellent data fits that are fully compatible with merozoite brood sizes and growth rates considered normal for *P. cynomolgi*. Thus, similar to predictions for its sister species *P. vivax* (reviewed in [2] and recently proposed by [10]), *P. cynomolgi* may also have a hidden reservoir of concealed iRBCs that results in the underestimation of the total parasite biomass during infection, and this convergent evidence provides additional support of the observation made in vivax malaria patients.

Interestingly, the modelling results indicate that concealment is quantitatively particularly important for iRBC populations early in the course of a blood-stage infection, specifically from the time the infection becomes patent until about day 19 after sporozoite inoculation. The contributions of concealed iRBCs later in the infection either correspond to a much lower percentage of the total iRBC population, or the peripheral parasitaemia becomes a more accurate representation of total iRBCs as host responses against the parasite begin to mature and prevent 100% invasion efficiency of released merozoites as assumed in the model, or inhibit putative host-parasite interactions required for productive concealment. However, there may also be a sub-population of iRBCs that multiply exclusively in the tissues as proposed by Baird [2], and future studies with careful analyses of *P. cynomolgi* or *P. vivax*-infected NHP tissues could address this directly. The purely computational outcome here is in line with the speculation that evading host removal is more critical for smaller iRBC populations that need to establish a blood-stage infection to prevent being eliminated by the host than for later populations that comprise overall higher parasitaemias. Similar observations suggesting possible cytoadherence and sequestration, or concealment, processes have been noted for the growth of *P. knowlesi*, which cycles every 24 h, and this has been observed in infections initiated by blood-stage inoculation (unpublished data). Such data would argue against an alternative hypothesis to explain the results of the current model, namely that primary liver-stage schizogony is not synchronous and merozoites may be released over a period of several days. Such asynchrony is unlikely, based on current data, but could warrant further careful investigation along with in-depth analysis using multiple methods to characterize the total parasite load during the critical days in question.

Although concealment may provide an early or late advantage during infection for the parasite, this process could have deleterious effects since locally increased parasite replication in the tissues could overwhelm the host by causing specific tissue damage. This damage could result in a cascade of uncontrolled pro-inflammatory responses and immunopathology, with advancement of an individual’s health towards the severe side of the malaria disease spectrum. Indeed, the modelling efforts undertaken here support such hypotheses as the parasite growth rate determined using the model of concealment early during infection correlates with severity of the infection in four out of five of the macaques in the cohort. The disease in these individuals had been described on a clinical basis as severe, non-severe or lethal [7]. Specifically, RIc14 and RSb14 were classified as non-severe and had inferred brood sizes of 39 and 27. In contrast, RFa14 and RFv13 had inferred brood sizes of 54 and 110, respectively, and irrecoverable lethal consequences required the humane euthanasia of RFv13, whose tissues showed severe pathology [60]. Therefore, it appears that the level of concealment, and thus the rate at which the parasites multiply early during infection, may correlate with disease presentation and be predictive of its progression. Indeed, the parasite replication rate has been demonstrated to be predictive of the progression of severe disease in *P. falciparum* [61], but comparable studies have not been performed with *P. vivax* and these are not readily feasible due to the lack of an in vitro culture system for this species [61]. The model developed here could be applied to data from *P. vivax* infection in susceptible NHP models, such as *Aotus* or *Saimiri* species [62], to help better evaluate if the parasite replication rate indeed generally correlates with clinical signs and symptoms of malaria. Such goals based on data from clinical studies with humans will be virtually impossible due to the need for longitudinal sampling and treatment of sick patients must take precedence. Additionally, a strategic future study to examine the tissue-specific parasite loads of *P. vivax* or *P. cynomolgi* to assess the total biomass in infected NHPs would be possible and highly informative.

In view of the complexities of known clinical pictures, and the multitude of host-parasite molecular and immunobiological interactions that may be relevant, concealment is not viewed here as the only factor that governs clinical presentation. In fact, RMe14 was classified as having severe disease but had an inferred brood size of 11, which is reasonable. Such a finding is intriguing and supports the likelihood for other factors to be important, including host genetics, as reported to be the case for patients with falciparum and vivax malaria [63, 64].

While the model presented here provides computational evidence that concealment may constitute an important process in *P. cynomolgi* infection and potentially during vivax malaria, the molecular mechanisms that are responsible for such processes require further study. As noted above, the *P. vivax* VIR proteins and their CYIR counterparts in *P. cynomolgi* comprise some initial candidates for adhesive interactions of the iRBCs that are in circulation. In support of a possible role of these proteins in pathogenesis, recent genome sequencing of natural *P. vivax* isolates revealed a much larger number (1200) of *pir/vir* genes than originally observed (346 [65]) and emphasizes the potential importance of this family for the parasite and possible immune evasion and pathogenesis mechanisms. The bone marrow has also gained attention recently as a possible, hypothesized, developmental niche for *P. vivax* iRBCs. Ex vivo studies showed that *P. vivax* merozoites, which have been known to invade reticulocytes, have a strong preference for invading a sub-population of reticulocytes that express high-levels of CD71, which are typically found in high densities in the bone marrow [36]. This work has ignited the hypothesis that *P. vivax* has evolved to invade these host cells in the bone marrow, as a protective mechanism to avoid the circulation, and that circulating iRBCs could also home to this location [36, 66]. These hypotheses can readily be tested using NHP model systems, such as the *P. cynomolgi* species in macaques and *P. vivax* in the New World monkey hosts [62].

## Conclusions

The model presented here may be a useful tool in the future to aid the quantification of the infected cell populations in NHP infection studies that can combine carefully timed blood and bone marrow draws, as well as necropsies, and also to identify the potential tissues in which the parasites may preferentially be concealed and make quantitative assessments of the parasite load *vis*-*à*-*vis* pathology. In 1991, Fremount and Rossan [67] found that the presence of *P. vivax* iRBCs was five to ten times greater in a juvenile marmoset compared to an adult, and that they predominated in liver, spleen and lung. Another study by these investigators, also from 25 years ago, provides an initial assessment of parasite counts in the tissues of a small cohort of *P. vivax*-infected *Saimiri* and *Aotus* monkeys [68]. The current model is not equipped to predict locations of concealed iRBCs in the macaques from this study, which was a 100-day longitudinal study followed by curative treatment. Nonetheless, this work shows that blood smears early in an infection may reflect much lower parasitaemias and pathologies than may actually be true, and it may be critical to recognize concealment in future research with humans and NHPs as a factor that may enhance successful parasitism.

Going forward, non-human primate models can be utilized to provide increasingly in-depth critical information that cannot be obtained from human infections. Mathematical models and experimental model systems, combined with modern experimental tools hold much potential, and such team efforts will predictably prove relevant for considering targeted interventions and aiming to benefit malaria elimination programmes.

## Additional file

**Additional file 1.** Details on data and models.
