# Supplementary material for: A model of Plasmodium vivax concealment based on Plasmodium cynomolgi infections in Macaca mulatta
Source: Malar J. 2017 Sep 18;16:375. doi: 10.1186/s12936-017-2008-4 (PMC5608162; doi:10.1186/s12936-017-2008-4)
Supplement: Supplementary file 1 — Additional file 1. Details on data and models. [file 12936_2017_2008_MOESM1_ESM.docx]

**Additional File: Details on Data and Models**

***A Model of Plasmodium vivax Concealment Based on***

***P. cynomolgi Infections in Macaca mulatta***

Luis L. Fonseca

Chester J. Joyner

MaHPIC Consortium

Mary R. Galinski

Eberhard O. Voit

**Details on Data and Models**

Details of the experimental infections of five rhesus macaques, data acquisition prior to and over a 100-day infection period, and raw data analysis were recently described in Joyner *et al*. [1]. The data from this experiment were deposited to PlasmoDB [2]. Selected highlights of the experimental design, clinical presentation of the animals, and data collection pertinent to the current modelling study are described below.

***1. Monkeys.*** The five rhesus monkeys (*M. mulatta*; RIc14, RSb14, RMe14, RFa14, and RFv13) used for this analysis were malaria-naïve males that were born and raised at the Yerkes National Primate Research Center at Emory University. The limitation to one sex eliminated potential blood loss issues associated with the female menstrual cycle, which could have been confounding data characterizing malarial anaemia. All experimental methods followed procedures and protocols approved by Emory’s Institutional Animal Care and Use Committee.

***2. Parasites.*** Experimental infections were initiated by intravenous inoculation of approximately 2,000 freshly isolated *P. cynomolgi* B strain sporozoites.

***3. Parasitaemia.*** Parasitaemia was determined by quantification of parasites utilizing thick and thin blood films as appropriate. A detailed explanation of the daily determination of parasitaemia is described in [1] and summarized here. Parasitaemia below 1% was determined using thick blood films whereas parasitaemias greater than 1% were determined using thin blood films. Parasitaemia was determined from thick blood films by quantifying the number of iRBCs per 500 leukocytes and then multiplying the ratio of the parasite count and the total number of leukocytes with the leukocyte count, which was determined by complete blood count analysis using an automatic cell analyzer (Beckman AcT-diff; Coulter Corporation). Parasitaemia determined from thin blood smears were determined by calculating the ratio between iRBCs and RBCs after counting 1000-2000 RBCs. The percent parasitaemia determined from this analysis was then multiplied by the RBC count obtained via the CBC to calculate parasites/µl.

***4. Growth Potential of P. cynomolgi.*** Like *P. vivax*, *P. cynomolgi* merozoites infect uninfected RBCs, grow and multiply inside these cells approximately every 48 hours, and release new broods of merozoites. The size range of such a brood for these related species is known to typically be about 16, with a normal range between 14 and 20 for different strains [3]. The maximum of this range determines an upper boundary for the growth potential of the parasite.

***5. Models.*** We designed two models to quantify the concealment of *P. cynomolgi* during macaque infections. Both models contain a pool representing iRBCs in circulation and a pool of iRBCs in concealment (Fig. S1). In Model 1, those parasites that had invaded RBCs in circulating blood proliferate and subsequently invade other RBCs in circulating blood, while those parasites that had infected RBCs in concealment proliferate and subsequently invade other RBCs in concealment; in addition, iRBCs may migrate between the two compartments in both directions. In Model 2, the parasites released by any iRBC migrate through the blood and infect RBCs in circulation. In both cases, the transport into concealment consists of two processes. One is directly proportional to the number of iRBCs in *B*, whereas the other is constant and thus independent of the number of iRBCs.

Analysis revealed that both models can fit the data. However, except for concealment in bone marrow, Model 1 would be somewhat difficult to explain, as release of merozoites could presumably not be contained within the vicinity of the releasing schizont, as the model suggests. Yet, in the bone marrow, large releases would easily overwhelm the pool of reticulocytes, thus leading to decreased reticulocyte numbers in the blood stream, for which other studies in our center do not provide evidence. Thus, Model 1 was abandoned and the focus turned to Model 2, which, in the text, is simply called “the model.”


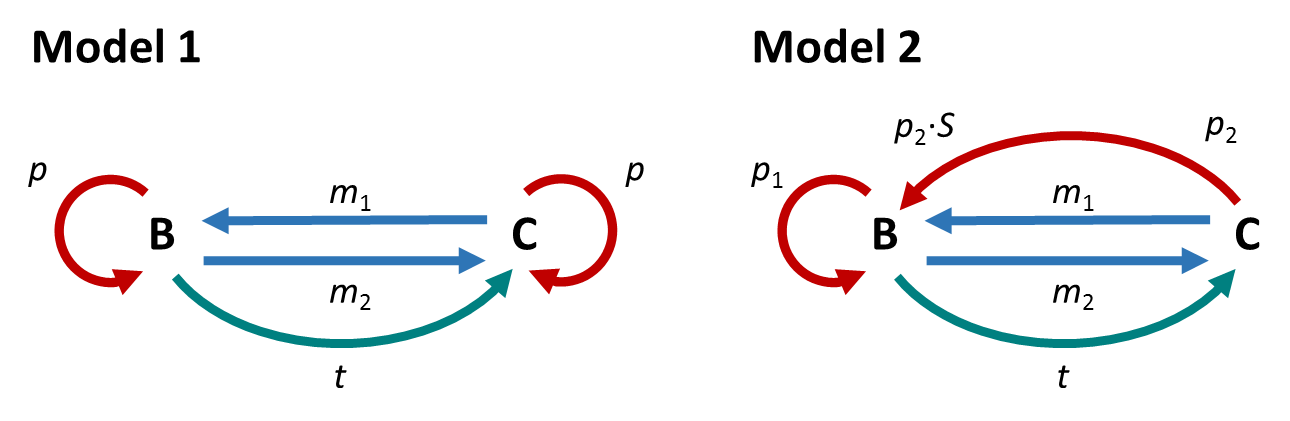


**Fig. S1. Two models accounting for iRBCs in the blood stream (B) or in concealment (C).** In Model 1, merozoites released from schizonts multiply mostly within the same compartment with a rate *p*, however, they may migrate between bloodstream and concealment with rates *m*_1_ and *m*_2_, respectively. Furthermore, there is constant net transport of cells into concealment with rate *t*. Model 2 is very similar, except that all merozoites released from any iRBCs enter the blood stream where they infect uninfected RBCs (uRBCs). Although the structure of the two models is similar, the rates of proliferation (*p*, *p*_1_, *p*_2_) are slightly different. In Model 1, each iRBC releases a brood of merozoites of size *S*, and these *S* merozoites almost immediately infect *S* uRBCs in the same location, either *B* or *C*. In Model 2, the same is true for the circulating pool *B*. However, each iRBC in *C* releasing merozoites disappears from pool *C*, and these merozoites yield *S* iRBCs in *B*.

***6. Limitations of the Model.*** The model uses differential equations, which represent gradual, average growth, whereas the actual growth process occurs in cycles as the iRBCs are maturing, interrupted by phases of no increases in numbers until the mature iRBCs are lysed and release their merozoite progeny. At the same time, *P. cynomolgi* infections, like in *P. vivax*, are not entirely synchronous over the course of a 2-day period, especially when they are initiated by sporozoites, as is the case in the current study. Also, the release of merozoites from the liver may occur over some period of time. As a consequence, multiple populations may be present simultaneously and mature on alternate days. In fact, the process may be close to a continuum, where some iRBCs from a given population mature, lyse the host cell, and egress earlier than others, and indeed, this has been demonstrated with *P. falciparum* [4]. It can be shown mathematically that such asynchrony leads to smoother, more ODE-like growth curves than entirely synchronous populations [5, 6]. Also, the model uses the same brood sizes, growth rates, parasite viability, and infectiveness throughout each analysis, whereas it is to be expected that merozoite brood sizes may exhibit variations from one parasite generation to the next. This simplifying assumption did not seem to compromise the validity of the model results, as we tested scenarios with different brood sizes and the results scaled essentially linearly. Moreover, the model assumes that all parasites are viable and able to infect and survive within each RBC successfully. Because this may not be entirely true, the growth rates are upper bounds of what is likely to happen. In spite of these limitations, the differential equations approximate the growth process in an intuitive and effective manner and are valid, as long as they are interpreted correctly. Of course one could perform simulations accounting for variations in brood sizes within the same monkey during the infection. However, such simulations did not seem warranted in the current study.

***7. Computation of the proliferation parameter p_2_.*** The rate of the proliferation processes *p*_1_ and *p*_2_ in Eq. (1) were assessed for a given brood size *S* of merozoites as follows, considering that a brood requires about two days to mature. Suppose that *Z* is the total number of iRBCs in *B* and *C* and that this number grows exponentially with a rate *r*, as it was observed (see Fig. 2) This rate is directly related to *S* as *r* = ln(*S*)/2, because *Z* satisfies the generic exponential function

$Z\left( T \right)=exp(a+r T)$, (2)

where *a* = 0 at time *T* = 0 and thus *Z*(0) = 1. Given that *P. cynomolgi* has a two-day asexual cycle and that *T* is measured in days, the function must satisfy the equation *Z*(2) = exp(0 + *r*·2) = *S*. Expressed in words, one parasite at an arbitrary time point 0 is replaced with *S* parasites at day 2. Taking logarithms yields *r* = ln(*S*)/2. Thus, considering that one iRBC is lost and *S* are gained in each cycle, and assuming 100% invasion efficiency, the growth can be written as the differential equation

$\dot{Z}=0.5 ln\left( S \right)\cdot Z$. (3)

Now consider the process of proliferation within *B* and *C* and the move of iRBCs from *C* into *B*, ignoring all other processes in the model. Suppose *C* is losing iRBCs through merozoite release with a rate *p*_2_. This loss, isolated from other processes, is given as

$\dot{C}= -p_{2} C$, (4)

where *p*_2_ is to be determined. For each iRBC lost from *C*, *S* as many parasites are produced, and these will infect uRBCs and become iRBCs in *B*. Furthermore, *B* loses iRBCs at the end of each 2-day growth and development cycle, but the emerging parasites more or less immediately infect uRBCs. If we call the combination of these two processes *f*(*B*), we obtain

$\dot{B}= p_{2}\cdot S\cdot C+f(B)$. (5)

The combined population of iRBCs thus grows according to the equation

$\dot{B}+ \dot{C}=p_{2}\cdot(S-1)\cdot C+f(B)$. (6)

By definition, the left-hand side equals $\dot{Z}$, so that we can use Eq. (3) and obtain

$0.5 ln\left( S \right)\cdot(B+C)=p_{2}\cdot(S-1)\cdot C+f(B)$. (7)

Sorting this term for *B* and *C* yields

$0.5 ln\left( S \right)\cdot B=- 0.5 ln\left( S \right)\cdot C+ p_{2}\cdot(S-1)\cdot C+f(B)$. (8)

Since we know that *f*(*B*) is a function of only *B*, the terms including *C* must combine to yield 0. The results are

$0.5 ln\left( S \right)=p_{2}\cdot\left( S-1 \right),$ (9)

$p_{2}=\frac{ln\left( S \right)}{2\cdot(S-1)}$, (10)

$f(B)=0.5 ln\left( S \right)\cdot B$. (11)

Taken together, the proliferation process, expressed in the size *S* of an average parasite brood, and for a 2-day cycle, is given as

$\dot{B}= \frac{ln\left( S \right)}{2\cdot(S-1)}\cdot S\cdot C+\frac{ln\left( S \right)}{2}\cdot B$ (12)

$\dot{C}= -\frac{ln\left( S \right)}{2\cdot(S-1)} C$ (13)

$\dot{Z}=\frac{ln\left( S \right)}{2} \cdot Z$ (14)

***8. Initial Values for B and C and Parameter Values.*** To obtain initial values for *B* and *C*, one day before iRBC detection was chosen as the initial time point *T*_0_. The growth data in Fig. 2 were fitted with exponential functions (*i.e*., per linear regression upon logarithmic transformation) and extrapolated back to one day before the first iRBCs had been detected, thus yielding *B*(*T*_0_). The ratio of *C*(*T*_0_) / *B*(*T*_0_) was considered as an additional free parameter. Thus, the model contains four free parameter values; three for the migration rates between *B* and *C*, as well as *C*(*T*_0_) / *B*(*T*_0_). These parameter values were optimized in a straightforward manner, using the MultiStart algorithm in Matlab. For additional confidence in the fits, the algorithm was initiated many times with different values to ensure that the solutions were robust. By thus optimizing the model with many initial guesses for all parameters, an entire ensemble of models was obtained that more or less fit the observed growth profiles of the parasite population equally well.

The optimal parameter values, leading to results in Figs. 3 and 4, are presented in Table S1. Interestingly, the first-order migration rates associated with *m*_1_ and *m*_2_ in the diagram of Model 2 (Fig. 1) are rather small. However, as first-order rate constants they need to be multiplied with the number of iRBCs to yield fluxes that are comparable with the transport parameter *t*. Thus, at day 15, the iRBC population of RSb14 has a size of about 8,000. The corresponding fluxes associated with *m*_1_, *m*_2_, and *t* are therefore 2.87, 0.0031, and 35.7. At day 17, fluxes associated with *m*_1_ and *t* are comparable. Early in the infection, however, the constant transport dominates.

For monkeys Rsb14 and Rlc14, whose parasite populations at day 11 are between 10 and 100, the values for *t* are about 36 and 60. By contrast, monkeys RFv13 and RFa14 have iRBC population sizes of about 200 and 800, and their values for *t* are between 500 and 1000. The implication is that a certain number of iRBCs constantly goes into concealment, but that this number depends on the severity of the infection.

**Table S1: Optimized Parameters used in Figures 3 and 4**

|  | **RSb14** | **RIc14** | **RFv13** | **RFa14** |
| --- | --- | --- | --- | --- |
| ***m*_1_** | 3.59 × 10^-5^ | 2.44 × 10^-14^ | 31.92 × 10^-3^ | 2.80 × 10^-14^ |
| ***m*_2_** | 3.91 × 10^-8^ | 3.20 × 10^-2^ | 7.04 × 10^-3^ | 3.18 × 10^-14^ |
| ***t*** | 35.7 | 60.6 | 548 | 1,000 |
| ***C*(0)/*B*(0)** | 2.06 | 6.34 | 13.8 | 4.96 |

Although the model is small, it is conceivable that quite different parameter settings could lead to data fits with similar residual errors. To analyze this possibility, a multi-start optimization scheme was used, and the optimization was reiterated with different initial guesses for all parameters many times (see *Methods*). The result for each monkey is therefore an ensemble of models that all fit the observed data similarly well. Fig. S2 shows results for monkey Rlc14; other results are similar. Since the model contains four parameters and the result of the optimization is a residual error, a visualization would require a five-dimensional representation. Instead, we set parameter *m*_1_ to zero, as it has a very small value for all satisfactory solutions, ran two Latin hypercube searches with 60,000 points each, and added to them results of randomly chosen points, which resulted in a total of 1,000,000 points. About 615,000 of these had no negative values, and among these we selected about 16,200 points that fell within 5% of the optimum of 0.7333. The best parameter combination (0, 4.7755×10^-4^, 59.48, 5.625) is indicated by a red dot and arrow. This point is very close, and has the same residual error, as the solution in Table S1, where *m*_1_=2.44 × 10^-14^. Surrounding this combination are excellent solutions (dark blue), which gradually become inferior (light blue, green, yellow), although they are all with 5% of the optimum. Importantly, the analysis did not detect far-away solutions of high quality.

**
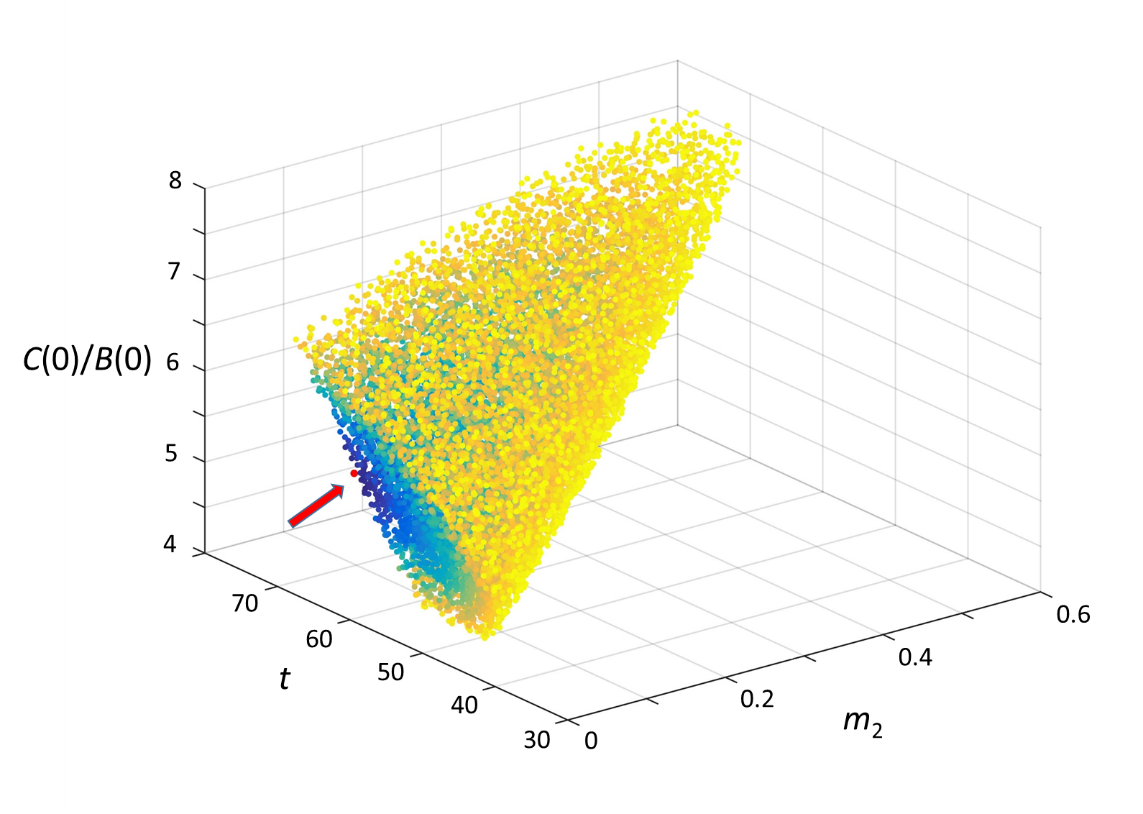
**

**Fig. S2. Ensemble of nearly optimal parameter values, which form a compact, localized set.** A combination of a Latin hypercube selection method and numerous additional optimizations with different, randomly chosen start values was performed. For *m*_1_ = 0, the parameter combinations with the smallest residual errors fill a domain within a 3-dimensional space, and the residual fitting error is shown in color, with dark blue dots being superior to light blue, green and yellow dots. Note that the yellow dots form a shell around a cone-like domain of better solutions. The results indicate that good solutions are clustered around the optimal solution (red dot and arrow).

1. Joyner C, Moreno A, Meyer EVS, Cabrera-Mora M, The MaHPIC Consortium, Kissinger JC, Barnwell JW, Galinski MR: ***Plasmodium cynomolgi* infections in rhesus macaques display clinical and parasitological features pertinent to modelling vivax malaria pathology and relapse infections.** *Malaria J* 2016, **15:**451.

2. PLlasmoDB: [**http://plasmodb.org/plasmo/mahpic.jsp**](http://plasmodb.org/plasmo/mahpic.jsp)**.** accessed 2017.

3. Coatney G, Collins W, Warren M, Contacos P: **The Primate Malarias.** Bethesda, Md.: U.S. National Institute of Allergy and Infectious Diseases 2003.

4. Reilly HB, Wang H, Steuter JA, Marx AM, Ferdig MT: **Quantitative dissection of clone-specific growth rates in cultured malaria parasites.** *Int J Parasitol* 2007, **37:**1599-1607.

5. Voit EO, Dick G: **Growth of cell populations with arbitrarily distributed cycle durations; I. Basic model.** *Mathem Biosci* 1983, **66:**229-246.

6. Voit EO, Dick G: **Growth of cell populations with arbitrarily distributed cycle durations; II. Extended model for correlated cycle durations of mother and daughter cells.** *Mathem Biosci* 1983, **66:**247-262.
